# Transcriptomic and physiological responses to fishmeal substitution with plant proteins in formulated feed in farmed Atlantic salmon (*Salmo salar*)

**DOI:** 10.1186/1471-2164-13-363

**Published:** 2012-08-01

**Authors:** Luca Tacchi, Christopher J Secombes, Ralph Bickerdike, Michael A Adler, Claudia Venegas, Harald Takle, Samuel AM Martin

**Affiliations:** 1 Institute of Biological and Environmental Sciences, University of Aberdeen, Tillydrone Avenue, Aberdeen, AB24 2TZ, UK; 2 BioMar Ltd, Grangemouth Docks, Grangemouth, FK3 8UL, UK; 3 AVS Chile S.A., Casilla 300, Puerto Varas, Chile; 4 Nofima, P.O. Box 5010, 1432, Aas, Norway

## Abstract

**Background:**

Aquaculture of piscivorous fish is in continual expansion resulting in a global requirement to reduce the dependence on wild caught fish for generation of fishmeal and fish oil. Plant proteins represent a suitable protein alternative to fish meal and are increasingly being used in fish feed. In this study, we examined the transcriptional response of Atlantic salmon (*Salmo salar*) to a high marine protein (MP) or low fishmeal, higher plant protein replacement diet (PP), formulated to the same nutritional specification within previously determined acceptable maximum levels of individual plant feed materials.

**Results:**

After 77 days of feeding the fish in both groups doubled in weight, however neither growth performance, feed efficiency, condition factor nor organ indices were significantly different. Assessment of histopathological changes in the heart, intestine or liver did not reveal any negative effects of the PP diet. Transcriptomic analysis was performed in mid intestine, liver and skeletal muscle, using an Atlantic salmon oligonucleotide microarray (Salar_2, Agilent 4x44K). The dietary comparison revealed large alteration in gene expression in all the tissues studied between fish on the two diets. Gene ontology analysis showed, in the mid intestine of fish fed PP, higher expression of genes involved in enteritis, protein and energy metabolism, mitochondrial activity/kinases and transport, and a lower expression of genes involved in cell proliferation and apoptosis compared to fish fed MP. The liver of fish fed PP showed a lower expression of immune response genes but a higher expression of cell proliferation and apoptosis processes that may lead to cell reorganization in this tissue. The skeletal muscle of fish fed PP vs MP was characterized by a suppression of processes including immune response, energy and protein metabolism, cell proliferation and apoptosis which may reflect a more energy efficient tissue.

**Conclusions:**

The PP diet resulted in significant effects on transcription in all the 3 tissues studied. Despite of these alterations, we demonstrated that high level of plant derived proteins in a salmon diet allowed fish to grow with equal efficiency as those on a high marine protein diet, and with no difference in biometric quality parameters.

## Background

Aquaculture production has been the fastest growing animal food-producing sector globally for over half a century, with production growing at an average rate of 8.1% per year since 1961 [1], representing almost 50% of human consumed fish [1,2]. Additionally, the capture from wild fisheries has plateaued with approximately three quarters of wild fisheries fished to capacity, overfished or recovering [3]. Atlantic salmon (*Salmo salar*) production alone now reaches over 1.5 million tonnes per year [4]. Total production in 2008 is estimated at 1.4 million tonnes, representing a 6% increase on 2007 [5]. The positive growth trend of the industry is expected to continue, reflecting the rising demand for healthy human food products high in protein and marine oils.

Piscivorous fish, which include salmonids, require high protein diets and until recently this was almost exclusively derived from wild caught non food fish such as anchovies and sardines. It has become apparent that with increasing fish meal demand due to the expanding aquaculture industry the proportion of protein in diets cannot be sustained by use of fish meal alone. During 2003, 20% of total fish meal usage for aquaculture feed production was utilized by the salmon industry [6], but continued development of diets has reduced the inclusion of fish meal in salmon diets from 500 g kg^-1^ to 350 g kg^-1^ by replacement with alternative protein sources [7]. Plant proteins currently represent the only economic and sustainable protein alternative to fish meal and are increasingly being used in commercial fish feed, with the most common being soybean meal (SBM) or soybean protein concentrate (SPC) which have a high protein content and contain the majority of essential amino acids required by salmonids. All plants contain a number of anti nutritional factors (ANFs) as part of their inherent defence mechanisms, effected by lectins, saponins, phytic acid and proteinase inhibitors amongst others [8]. In early attempts to use plant proteins in fish diets ANFs were often co purified with the proteins, leading to metabolic dysfunction in fish liver [9], inflammation in the intestine [10,11], reduced protein deposition [12], and an impaired immune response [13]. Currently used plant replacement diets use a combination of different plant proteins, with SPC often the major component [14]. Processing of soy protein to SPC with alcohol-extraction removes the majority of ANFs facilitating use of these products in fish feeds at a high inclusion level without causing enteritis or other gross morphological changes [15]. Replacement of up to 50% of the fish meal with a mixture of plant proteins is possible in rainbow trout without affecting fish growth, and even complete replacement of fish meal with SPC has been reported [16,17]. Other successful plant proteins used regularly in aquaculture include wheat gluten at up to 40% of fish meal replacement in feed for salmon and trout (Hardy 1996), corn gluten and sunflower meal at up to 30% replacement, which show no adverse effects on salmonid growth performance [18-20]. Extensive studies investigating responses of Atlantic salmon to such diets indicate that a replacement of fish meal close to 100% can be used without negative effects on salmon growth providing the proteins are highly purified and the amino acid profile is well balanced [21]. High levels of pea protein concentrate do still cause enteropathy in salmon though [22].

In this paper we report a whole transcriptome based study on post-smolt Atlantic salmon fed on a low marine protein diet, where marine protein fish meal was partially replaced with a combination of plant derived proteins, compared to fish fed a diet high in fish meal. The high plant protein (PP) diet contained a balanced combination of soy protein concentrate, corn gluten and wheat gluten, and was supplemented with lysine and methionine to ensure the requirement for essential amino acids was met. The fish were fed for 77 days following which growth, feed efficiency and quality biometric parameters were measured and tissues were collected for histopathological changes and gene transcription analysis. The transcriptional response was examined using an Atlantic salmon“salar_2” microarray platform (Agilent) [23], enriched for genes involved in protein metabolism, lipid metabolism and immune function.

## Results

### Feeding trial

During the 77 day feeding trial fish were fed either a high marine protein (MP) diet or a plant protein replacement (PP) diet (Table 1), and both groups doubled in weight over this period. No significant differences were seen between the groups in growth performance, feeding efficiency, condition factor or organ indexes (Table 2). There were also no significant mortalities in any of the tanks.

**Table 1:** Dietary formulations

	**PP**	**MP**
Raw Materials (% of inclusion)		
Fish meal (%)	26.0	51.0
Fish oil (%)	19.2	17.3
Wheat (%)	6.3	11.9
Maize gluten (%)	10.0	3.0
Sunflower meal (%)	15.0	10.0
Rapeseed meal (%)	3.0	3.0
Wheat gluten (%)	1.3	
Soy protein concentrate(%)	18.0	3.0
Premix (%)	0.4	0.4
Monocalcium phosphate (%)	0.5	0.3
Lysine (%)	0.3	
Methionine(%)	0.1	
Proximate Analysis (% in feed)		
Protein (%)	44.8	44.3
Oil(%)	23.5	24
Ash(%)	6.8	8.9
Moisture(%)	7.7	8.5
Marine protein (as % of total Crude Protein)	39%	78%

Dietary formulation of high marine protein diet (MP) and plant protein diet (PP). Values represent percentage inclusion or content. Marine content calculated for total protein. The Fish oil is a commercial Chilean fish oil.

**Table 2:** Growth performance and somatic indexes

	**PP**		**MP**	
	**Mean**	**+/− SD**	**Mean**	**+/− SD**
Initial Weight (g)	110.6	0.8	110.8	0.4
Final Weight (g)	231.4	1.9	237.0	3.2
SGR (%)	1.09	0.02	1.12	0.02
FCR	0.87	0.01	0.90	0.03
SFR (%)	0.95	0.02	1.00	0.04
Condition factor	1.08	0.00	1.06	0.03
CSI	0.08	0.00	0.08	0.01
HSI	1.09	0.07	1.06	0.02
ISI	2.32	0.20	2.40	0.20

Growth performance and somatic indexes. SGR represents the specific growth rate of fish, FCR is the feed conversion ratio, SFR is the specific feeding rate, CSI is the cardiac somatic indice, HSI is the hepatic somatic indice and ISI is the intestinal somatic indice.

### Histology

A number of histological changes were found, however different types of anomalies were observed in tissues of individuals from both treatment groups and such changes were observed in low frequency indicating that the fish were in a good condition (Table 3). There was no significant dietary effect for any histological change except the frequency of perivascular and peri‐biliar duct infiltrations in the liver, where 21% of fish fed the MP diet exhibited this change compared to 3.8% of fish fed the PP diet (Table 3). A number of fish from both diets showed a mild fatty change in the liver, but this was not restricted to a particular dietary group (Table 3). Equally, some anomalies in heart were observed at a low level in both dietary treatments. For the intestine, no changes were observed in pyloric caeca or mid intestine, whereas for the distal intestine an inflammatory infiltration in the submucosa and lamina propria was observed but at a low level in both diets, with no significant difference in occurrence between dietary groups (Table 3).

**Table 3:** Histopathological observations/changes

**Organ**	**Changes**	**PP**	**MP**	*P*-value*
Liver	No significant findings	39.0%	33.3%	0.39
	Mild fatty change	38.1%	31.4%	0.312
	Mild to moderate fatty change	8.6%	7.6%	0.801
	Moderate fatty change	8.6%	8.6%	n.d.
	Moderate to severe fatty change	0.0%	1.9%	0.156
	Severe fatty change	0.0%	0.0%	n.d.
	Perivascular infiltration	2.9%	6.7%	0.196
	Peri biliar ducts infiltration	3.8%^a^	21.0%	<0.001
	Necrotic foci w/infiltration	1.0%	0.0%	0.317
	Mild congestion	0.0%	1.0%	0.317
	Focal necrosis	0.0%	2.9%	0.082
	Mild sinusoidal dilatation	0.0%	1.0%	0.317
	Biliar ducts esclerosis	0.0%	2.9%	0.082
	Biliar ducts proliferation	0.0%	0.0%	n.d.
	Foci of inflammatory cells	0.0%	1.0%	0.317
Heart (ventricle)	No significant findings	99.0%	98.1%	0.562
	Severe infiltration and myodegeneration	1.0%	1.0%	n.d.
	Infiltration, no myodegeneration	0.0%	1.0%	0.317
	Presence of melanin granules	0.0%	0.0%	n.d.
Mid-intestine and Pyloric caeca	No significant findings	100%	100%	n.d.
Distal intestine	No significant findings	97.1%	95.2%	0.472
	Infiltration of submucosa and lamina propria, widened of lamina propria within folds (simple and complex), prominent goblet cells/low number of supranuclear vacuoles	2.9%	4.8%	0.472

Histopathological observations/changes. Values (in percentage) represent occurrence where *n* = 105. Significantly different when P < 0.05, different superscript letters indicate significant differences within each row-wise comparison.

### Transcriptomic responses

Fish fed the PP diet showed changes in transcriptome in all 3 tissues examined compared to fish fed the marine protein rich diet MP. In total 8,151 oligo features were found to be expressed at different levels as a result of the dietary manipulation. For subsequent analysis only significant (*p* < 0.05) features with a fold difference of greater than 2 were analysed further (1961 mRNAs). The intestine exhibited the greatest differential gene expression with 1,236 genes significantly different in expression level between fish fed the PP diet and MP diet. Of these 615 genes were higher in expression and 621 lower in PP fed fish. In skeletal muscle 505 genes had an expression higher than two fold with 132 higher in PP fed fish and 373 lower and in liver 220 genes were found modified with 161 higher and 59 genes lower in PP fed fish vs MP fed fish (Table 4).

**Table 4:** Genes significantly different in mid intestine of fish fed PP diet

**Probe name**^1^	**ACC**^2^	**FC ± SEM**^3^	**Identity**^4^
**Immune function and stress related**			
Ssa#CK897125	CK897125	7.6 ± 1.1	B-cell linker
Ssa#CL60Contig3	X70167	5.2 ± 1.2	MHC class II antigen beta chain
Ssa#STIR13675	TC71772	5.0 ± 1.3	CD209f
Ssa#NP9934055	NP9934055	4.8 ± 1.4	T cell receptor alpha
Ssa#STIR21272	TC82967	4.3 ± 1.2	Interferon inducible mx protein
Ssa#S35685629	S35685629	4.3 ± 1.0	TCR-gamma
Ssa#S30239635	S30239635	2.8 ± 1.2	IRF1
Ssa#STIR10385	TC67231	2.5 ± 1.1	Vig-2 protein
Ssa#STIR15805	TC74805	2.4 ± 1.0	cd3 epsilon
Ssa#STIR05606	BT056756	2.4 ± 1.1	β2 microglobulin
Ssa#gi156446662	EF579742	2.0 ± 1.2	MyD88
Ssa#CL81Contig1	BT072778	2.0 ± 1.1	Virus induced TRIM protein
Ssa#S37438814	S37438814	2.0 ± 1.0	CD3 gamma/delta
Ssa#STIR00071_2	DW580947	2.0 ± 1.2	Interferon induced protein 35
Ssa#S18833713	S18833713	−7.5 ± 1.2	Serum lectin 2
Ssa#CK882427	CK882427	−3.2 ± 1.3	Serum amyloid A
Ssa#S35474845	S35474845	−3.0 ± 1.1	HSP β-7
Ssa#S35677496	S35677496	−2.9 ± 1.1	CCR3
Ssa#S31986130	S31986130	−2.7 ± 1.2	Macrophage colony stimulating factor receptor
Ssa#S18892409	S18892409	−2.7 ± 1.1	IgM
Ssa#STIR03818	NM_001141099	−2.4 ± 1.3	CXC13
Ssa#STIR19205	TC79827	−2.3 ± 1.1	HSP 70a
Ssa#STIR13083	TC70912	−2.3 ± 1.2	SAMHD1
Ssa#S35663823	S35663823	−2.2 ± 1.2	Complement c1q
Ssa#S35583279	S35583279	−2.1 ± 1.0	IL-17D
Ssa#S35517748	S35517748	−2.0 ± 1.1	HSP 30
Ssa#S35536386	S35536386	−2.0 ± 1.1	HSP β-11
Omy#gi31087931	AY160984	−2.0 ± 1.2	IL-8
Ssa#TC106255	TC106255	−2.0 ± 1.1	Galectin-4
**Cell proliferation and apoptosis**			
Ssa#S35582566	S35582566	7.1 ± 1.2	Caspase-14
Ssa#CL201Ctg1	NM_001139921	2.4 ± 1.0	Caspase-3
Ssa#S35693335	S35693335	2.2 ± 1.0	Caspase 8
Ssa#S32005165	S32005165	−9.4 ± 1.0	DNA topoisomerase 2-alpha
Omy#S34421775	S34421775	−6.6 ± 1.5	Replication factor C subunit 3
Omy#CA346576	CA346576	−4.5 ± 1.2	Tetraspanin-14
Ssa#CA038824	CA038824	−3.9 ± 1.1	Fgfr3 protein
Omy#S34313679	S34313679	−3.6 ± 1.4	Cyclin A1
Omy#S22901990	S22901990	−3.4 ± 1.0	Chromodomain-helicase-DNA-binding protein 7
Ssa#S47728937	S47728937	−3.1 ± 1.1	Tetraspanin-16
Ssa#S35496360	S35496360	−2.9 ± 1.0	DNA-repair protein complementing XP-A cells
Ssa#S35490761_S	S35490761	−2.7 ± 1.1	Transcription factor HES-1
Ssa#STIR38390	TC108636	−2.7 ± 1.3	RNA helicase
Ssa#S35531441	S35531441	−2.4 ± 1.1	Melanoma-derived growth regulatory protein
Ssa#STIR17200	TC76867	−2.4 ± 1.0	Tumor necrosisalpha-induced protein 2
**Protein metabolism**			
Ssa#S35499913	S35499913	6.5 ± 1.0	Titin
Ssa#S31974046	S31974046	4.2 ± 1.1	Cathepsin C
Ssa#S30293144	S30293144	3.9 ± 1.5	Proteasome subunit alpha type-5
Ssa#STIR05302	BT046757	3.2 ± 1.1	Proteasome subunit beta type-7
Ssa#CL233Ctg1	BT072668	3.0 ± 1.0	Cathepsin Z
Omy#S15290109	S15290109	3.0 ± 1.2	Keratin e1
Ssa#STIR25562	TC89420	3.0 ± 1.1	Troponinskeletal muscle
Ssa#STIR20536	TC81827	2.9 ± 1.1	Cathepsin A
Ssa#STIR24947	TC88495	2.8 ± 1.2	Keratin 18
Ssa#DY714088	DY714088	2.7 ± 1.0	Ribosomal protein S6 kinase b
Ssa#KSS4531	KSS4531	2.6 ± 1.1	Ubiquitin-conjugating enzyme E2
Ssa#STIR08978	TC65424	2.2 ± 1.0	Eukaryotic translation initiation factor 4e
Ssa#DY712052	DY712052	2.0 ± 1.1	Gamma-tubulin complex component 4
Ssa#S30279979	S30279979	2.0 ± 1.2	Eukaryotic translation initiation factor 2 subunit 1
Ssa#S30240560	S30240560	2.0 ± 1.2	β-actin
Ssa#KSS1565	KSS1565	2.0 ± 1.0	Proteasome subunit alpha type-6
Ssa#KSSb2668	KSSb2668	2.0 ± 1.2	Tubulin beta-1 chain
Ssa#STIR39880	TC110797	2.0 ± 1.1	Proteasome subunit alpha type-7
Ssa#S31996856	S31996856	2.0 ± 1.1	E3 ubiquitin-protein ligase RNF128
Ssa#STIR19643	TC80514	−3.9 ± 1.3	Myosin light chain 1–3
Ssa#TC76471	TC76471	−3.6 ± 1.2	Tropomyosin-1 alpha chain
Ssa#AJ425777	AJ425777	−2.7 ± 1.3	60 S ribosomal protein L34
Ssa#STIR11900	TC69277	−2.5 ± 1.1	Myosin ic
Ssa#S35582711	S35582711	−2.3 ± 1.0	Tropomyosin-1 alpha
Ssa#S35505113	S35505113	−2.2 ± 1.1	60 S ribosomal protein L30
Ssa#FC072705	FC072705	−2.1 ± 1.1	40 S ribosomal protein S10
Omy#CX150249	CX150249	−2.1 ± 1.0	60 S ribosomal protein L36
**Lipid metabolism**			
Ssa#CB509140	CB509140	25.3 ± 1.2	Fatty acid-binding protein
Ssa#S31963508	S31963508	14.5 ± 1.5	Apolipoprotein A-I
Ssa#STIR00045_2	AY170327	5.2 ± 1.0	PUFA elongase 5A
Ssa#STIR00100_2	CK887422	4.7 ± 1.0	Delta-6 fatty acyl desaturase
Omy#S18154618	S18154618	4.4 ± 1.2	Glycolipid transfer protein
Ssa#STIR00093_4	AF478472	4.3 ± 1.1	Delta-5 fatty acyl desaturase
**Energy and mitochondrial activity/kinases**			
Ssa#STIR00144_4	TC64612	4.2 ± 1.0	Glyceraldehyde-3-phosphate dehydrogenase
Ssa#STIR39924	TC110855	2.6 ± 1.2	Cytochrome P450
Ssa#S32006874	S32006874	2.3 ± 1.1	Peroxisomal membrane protein 11 C
Ssa#S30263228	S30263228	2.4 ± 1.1	Succinate dehydrogenase
Ssa#S35661441	S35661441	2.0 ± 1.0	Fructose-1.6-bisphosphatase 1
Ssa#S32000347	S32000347	−2.5 ± 1.2	Hemoglobin subunit beta-1
**Cellular transport**			
Ssa#S30284157	S30284157	23.6 ± 1.2	Solute carrier family 22
Ssa#STIR21606	C83482	9.1 ± 1.2	Solute carrier family 16
Ssa#DW575876	DW575876	7.2 ± 1.3	Solute carrier family 39
Ssa#STIR19539	TC80341	4.6 ± 1.0	Solute carrier family 31
Ssa#STIR19539	S35536215	2.8 ± 1.0	Solute carrier family 25
Ssa#S35454393	S35454393	2.1 ± 1.1	Solute carrier family 27
Omy#TC172518	TC172518	−2.2 ± 1.1	Beta globin
Ssa#CK991045	CK991045	−2.0 ± 1.0	Hemoglobin subunit alpha

There were no genes expressed at different levels in common between all the tissues examined indicating there was not a systematic response to the diets. However there was a co regulation of features between the mid intestine and skeletal muscle with 9 genes higher in both tissues of PP fed fish vs MP fed fish and 10 lower in both. Similarly in the liver and intestine 6 genes were found to be significantly different in common, with 5 genes higher in expression and 1 gene lower in the PP fed fish. To further investigate the biological significance of the differently expressed gene sets we used gene ontology analysis to help identify biological processes that were significantly different in tissues following the feeding trial.

**Figure 1:**
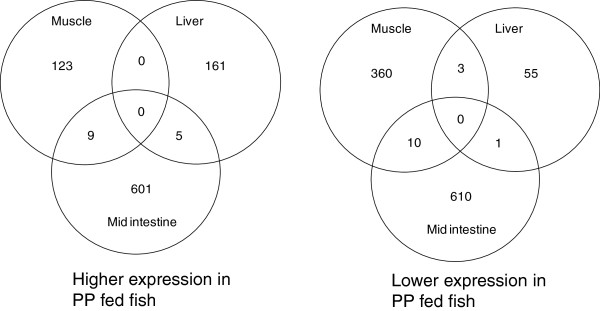
**Venn diagram showing numbers of genes identified as expressed at different levels by microarray analysis.** Summary of numbers of genes higher and lower expressed. The genes presented are all significantly different in expression (p < 0.05) with >2 fold change in expression.

Annotation of the microarray allowed 77% of the features to be allocated to a functional protein and 55% of these proteins were assigned a gene ontology (GO) identifier. This allowed statistical analysis for enrichment for GO biological processes to help interpret the changes in the transcriptome following the feeding trial (Figure 1) to gain a holistic view of which biological processes were significantly modified following feeding the PP diet.

### Genes expressed at different levels in mid intestine

The intestine is the major tissue to come into direct contact with any feed components and for this reason may be very sensitive to dietary changes. This is shown here by the intestine having the greatest transcriptional response in terms of the number of genes expressed at different levels and the magnitude of different expression of these genes. Several biological processes were significantly different in the PP diet fed fish, related to intestinal functions including immune and stress related processes, protein metabolism, energy and mitochondrial activity, lipid metabolism and transport (Table 4).

#### Immune and stress response

Genes encoding proteins related to both innate and acquired immune function were expressed at different levels by the dietary treatments. For fish fed PP innate immune parameters were found to be both higher and lower in expression indicating a dynamic response. Modulators of the immune response were higher in PP fed fish, such as MyD88, a key transcription factor associated with induction of an inflammatory response and P105 subunit of an inhibitory protein that sequesters NF-*κ*B in the cytoplasm. Another transcription factor, interferon regulatory factor (IRF) 1 was also higher, and potentially increased expression of interferon responsive genes including Mx, Vig-2, interferon induced protein 35 and virus induced TRIM protein. Genes encoding cytokine receptors (IL-17R) and regulators of cytokine function including the suppressor of cytokine signalling (SOCS)-7) were also higher. A number of genes related to innate immune responses were also lower, and included IL-17D, MCSFR and chemokines such as CCL3, CXC13 and IL-8 (CXCL8). Other innate immune serum proteins were also lower in expression including serum amyloid A, and a number of lectins. Genes related to proteins in the acquired immune response were mostly higher in expression in fish fed the PP diet compared to the MP diet. For example, T cell receptor chains (α and γ) and their signalling subunits CD3 epsilon and gamma/delta were higher as were genes involved with antigen presentation such as β2 microglobulin and MHC class II). Related to antibody production by B cells, a B-cell linker protein that regulates B-cell function and development was higher in PP fed fish as was a B cell enhancing factor.

Regarding stress related genes, PP fed fish showed an elevated expression of glutathione-S-transferase and a thioredoxin interacting protein, involved in reducing oxidative stress, while heat shock proteins (HSP) β-7, β-11, 30 and 70a were all suppressed.

#### Cell proliferation and apoptosis

The intestinal transcription of genes encoding proteins related to cell proliferation (tumor necrosis alpha-induced protein 2, tetraspanins-14 and −16 and melanoma-derived growth regulatory protein) and the cell cycle (DNA topoisomerase 2-alpha, replication factor C subunit 3, FGFR3 protein, cyclin A1) were found to be higher in PP fed fish; whilst transcription of caspases 3, 8 and 14,which are directly involved in apoptosis, were found to be lower in expression relative to fish fed MP.

#### Protein metabolism

Feeding the PP diet the expression of genes involved in protein metabolism was significantly different compared to MP. For example, protein synthesis related genes including elongation factor, translation initiation factors 2 and 4e and ribosomal protein S6 kinase b were higher, while a number of 60 S ribosomal protein encoding genes were lower relative to MP fed fish. Protein degradation encoding genes were more highly expressed as shown by lysosomal proteases, cathepsins A, C, and Z, ubiquitin proteasome route (UbP) of proteolysis, and proteasome subunits α5, α6, α7 and β7. Genes involved in targeting of proteins for degradation in the UbP were also higher including ubiquitin conjugating enzyme E2 and ubiquitin E3 ligases. Genes encoding the structural proteins troponin, tubulin, titin, actin and keratin were all found to have a higher expression level whereas a number of myosin mRNAs were lower in expression.

#### Lipid metabolism

A higher transcript level of genes involved in lipid transport and metabolism was observed, with higher transcription of fatty acid-binding protein, apolipoprotein A-I, apolipoprotein B and delta-6 fatty acyl desaturase, delta-5 fatty acyl desaturase, PUFA elongase 5A and glycolipid transfer protein, respectively.

#### Energy and mitochondrial activity/kinases

There was an induction of many genes related to energy metabolism in pathways such as glycolysis (glyceraldehyde-3 phosphate dehydrogenase), the tricarboxylic acid cycle (succinate dehydrogenase) and gluconeogenesis (fructose 1-6- bisphosphate) which were expressed at a higher level in PP fed fish relative to the MP fed fish.

#### Cellular transport

Transport of solutes into and from a cell is an energy demanding process. Genes involved in membrane transport were higher in expression in fish fed the PP diet, and included a number of solute carrier family members such as SCF22 (organic cation transporter), SCF 27 (fatty acid transporter), SCF 25 (mitochondrialphosphate carrier), SCF 39 (zinc transporter), SCF 16 (monocarboxylic acid transporter), and SCF 31 (copper transporter). Two genes encoding haemoglobin subunits (hemoglobin subunit alpha-4 and hemoglobin subunit beta-1) and beta globin, however, were found to have a lower relative expression level.

### Genes expressed at different levels in liver

Several biological processes were significantly different in liver that can be related to immune parameters, stress responses, and protein and lipid metabolism, with key genes shown in Table 5.

**Table 5:** Genes significantly different in liver of fish PP diet

**Probe name**^1^	**ACC**^2^	**FC ± SEM **^3^	**Identity**^4^
**Immune function and stress response**			
Ssa#S35601811	S35601811	2.7 ± 1.0	MPV17 protein
Ssa#gi156446662	EF579742	2.4 ± 1.1	MyD88
Omy#S18150823	S18150823	2.2 ± 1.1	Amine oxidase
Ssa#STIR13675	TC71772	2.0 ± 1.0	CD209f
Ssa#CK874360	CK874360	2.0 ± 1.1	Heat shock 70
Ssa#STIR36546	TC105929	−2.8 ± 1.1	C-type MBL-2 protein
Ssa#NP797925	NP797925	−2.4 ± 1.1	MHC class I alpha 2
Ssa#STIR04816	NM_001140849	−2.4 ± 1.2	Hepcidin
Ssa#NP9934311	NP9934311	−2.2 ± 1.1	T cell receptor alpha
Ssa#S48440415	S48440415	−2.2 ± 1.3	SMAD3
Ssa#S35663823	S35663823	−2.1 ± 1.1	Complement c1q
**Cell proliferation and apoptosis**			
Ssa#CA041082	CA041082	2.6 ± 1.2	Transforming growth factor beta receptor
Ssa#S35486979	S35486979	2.5 ± 1.0	Cell death activator CIDE-3
Ssa#S35559076	S35559076	2.3 ± 1.0	Angiopoietin-related protein 4
Ssa#S30276711	S30276711	2.3 ± 1.1	Activin receptor type-1B
Ssa#S35582821	S35582821	2.3 ± 1.0	Serine protease HTRA1
Ssa#STIR31305	TC98147	2.2 ± 1.1	Annexin A3
Omy#S18150823	S18150823	2.2 ± 1.0	Amine oxidase
**Protein metabolism**			
Ssa#DW574268	DW574268	2.4 ± 1.1	Ribosomal protein S18
Ssa#STIR03071	BT048999	2.3 ± 1.1	Proteasome beta type 3
Ssa#KSSb2684	KSSb2684	2.2 ± 1.2	Peptidyl-prolyl cis-trans isomerase
Ssa#STIR16404	TC75662	2.2 ± 1.1	Calpain
Ssa#STIR04151	NM_001141015	2.1 ± 1.0	Ribosomal protein l39

#### Immune and stress response

Genes encoding proteins involved in innate immunity such as complement c1q, C-type MBL-2 and a dendritic cell specific lectin CD209f which binds mannose carbohydrate molecules, and hepcidin a major liver associated antimicrobial peptide were lower in PP fed fish. A limited number of genes related to adaptive immunity were also found to be expressed at different levels, with MHC class I and SMAD 3 lower in expression level, whereas MyD88 was higher in fish fed the PP diet relative to MP fed fish. Moreover, genes involved in the oxidative response (MPV17 protein, amine oxidase and heat shock cognate 70 kDa protein) were also higher in fish fed the PP diet.

#### Cell proliferation and apoptosis

The PP diet stimulated/modified hepatic cell turnover as indicated by higher expression of serine protease HTRA1 and annexin A3 and the stimulation of apoptotic processes through genes involved in TGF beta pathways (cell death activator CIDE-3 and angiopoietin-related protein 4).

#### Protein metabolism

Hepatic protein metabolism was clearly stimulated in PP fed fish since all genes significantly different in expression were higher in these fish, including ribosomal protein S18a, ribosomal protein l39 and peptidyl-prolyl cis-trans isomerise. Similarly genes involved in protein degradation were more highly expressed as seen with the non-lysosomal protein calpain and the proteasome subunit β3.

### Genes expressed at different levels in skeletal muscle

The GO analysis showed a number of processes to be significantly different in skeletal muscle that can be related to protein metabolism, immune function and energy metabolism (Table 6).

**Table 6:** Genes significantly different in skeletal muscle of fish PP diet

**Probe name**^1^	**ACC**^2^	**FC ± SEM**^3^	**Identity**^4^
**Immune function**			
Ssa#STIR12634	TC70300	5.0 ± 1.2	Vig-2
Ssa#STIR00067_2	U66477	3.2 ± 1.3	Interferon inducible Mx protein
Ssa#CK894557	CK894557	−11.2 ± 1.4	MHC class Ib antigen
Ssa#STIR00132_2	NM_001140254	−7.4 ± 1.3	Tumor necrosis alpha-induced protein 2
Omy#NP565601	NP565601	−6.4 ± 1.4	T-cell receptor beta
Ssa#S31982089	S31982089	−5.4 ± 1.1	Platelet-activating factor receptor
Ssa#STIR12498	TC70105	−4.6 ± 1.2	γ-ip (CXCL10)
Ssa#S48440415	S48440415	−3.4 ± 1.1	SMAD3
Omy#S15331473	S15331473	−3.2 ± 1.2	Interferon-inducible protein Gig2-like
Ssa#S35544087	S35544087	−3.1 ± 1.3	B-cell CLL/lymphoma 7 protein family member B
Ssa#STIR13083	TC70912	−3.6 ± 1.0	SAMHD1
Omy#S18153399	S18153399	−2.4 ± 1.1	CD80
Ssa#KSS5035	KSS5035	−2.2 ± 1.2	Interferon regulatory factor 2-binding protein
Ssa#STIR00071_3	DW580947	−2.3 ± 1.1	Interferon -induced protein 35
Ssa#STIR08451	TC64790	−2.2 ± 1.2	Heat shock protein 47
Ssa#S35480903	S35480903	−2.0 ± 1.2	Heat shock protein 30
Ssa#STIR29454	TC95297	−2.1 ± 1.1	Beta defensin 1
**Cell proliferation and apoptosis**			
Ssa#S35602638	S35602638	3.7 ± 1.4	Tripartite motif 39
Ssa#TC110067	TC110067	3.5 ± 1.5	Cell death inducing protein
Ssa#STIR08668	TC65039	3.1 ± 1.1	Bh3 interacting domain death agonist
Ssa#STIR12507	TC70118	3.0 ± 1.3	Syndecan 4
Ssa#STIR02208	BT049868	−38.6 ± 1.2	Caspase 14
Ssa#S35559333	S35559333	−4.1 ± 1.3	Ankyrin repeat domain-containing protein 54
Omy#TC151190	TC151190	−2.9 ± 1.2	Nucleostemin
Ssa#S35550715	S35550715	−2.8 ± 1.2	Growth hormone-inducible transmembrane protein
Ssa#S35585784	S35585784	−2.6 ± 1.1	Caspase-8
Ssa#KSS5035	KSS5035	−2.4 ± 1.2	Interferon regulatory factor 2-binding protein 2-B
**Protein metabolism**			
Ssa#S35682089	S35682089	4.2 ± 1.3	Ubiquitin-like protein 1
Ssa#STIR17445	TC77227	2.6 ± 1.3	Acta1 protein
Ssa#STIR21465	TC83266	2.5 ± 1.2	Actin-binding
Ssa#STIR03710	BT048358	2.0 ± 1.2	Proteasome subunit beta type-9
Ssa#STIR00115_4	BT045917	2.0 ± 1.2	Tropomyosin-1 alpha chain
Ssa#S32008331	S32008331	−3.6 ± 1.7	40 S ribosomal protein S16
Ssa#CL224Ctg1	NM_001140473	−2.8 ± 1.5	Receptor-interacting serine/threonine-protein kinase 4
Omy#CX150460	CX150460	−2.7 ± 1.1	60 S ribosomal protein L27
Omy#S15320960	S15320960	−2.6 ± 1.1	Cathepsin D
Ssa#TC111443	TC111443	−2.5 ± 1.1	Serine/threonine-protein kinase 35
Ssa#FC072705	FC072705	−2.3 ± 1.0	40 S ribosomal protein S10
Ssa#S35583213	S35583213	−2.1 ± 1.2	Serine/threonine-protein kinase PINK1
Ssa#S35591236	S35591236	−2.1 ± 1.1	Histidine triad nucleotide-binding protein 3
**Energy metabolism**			
Ssa#STIR39924	TC110855	2.5 ± 1.1	Cytochrome P450
Ssa#S32006874	S32006874	2.2 ± 1.1	Peroxisomal membrane protein 11 C
Ssa#KSS5038	KSS5038	−4.0 ± 1.3	Phosphoglycerate kinase
Ssa#STIR19484	TC80264	−3.1 ± 1.3	Cytochrome c oxidase polypeptide viii
Ssa#CL200Ctg1	BT059338	−2.8 ± 1.3	L-lactate dehydrogenase B chain
Ssa#S30282925	S30282925	−2.7 ± 1.1	Peroxisomal 3.2-trans-enoyl-CoA isomerase
Omy#CX150319	CX150319	−2.4 ± 1.2	Cytochrome c oxidase subunit II
Ssa#STIR13627	TC71700	−2.1 ± 1.2	Cox18 cytochrome c oxidase
Ssa#EL698167	EL698167	−2.0 ± 1.1	Malate dehydrogenase 1

List of selected mRNAs found to be higher or slower in expression following the feeding trial in mid intestine (a), liver (b) or skeletal muscle (c) and grouped according to functional classes (shown in bold). The selection was based on manual assignment of function and genes with greatest fold differences in expression are presented, the genes that are lower in expression are denoted by (−) value. The genes shown were significant at p < 0.05 following t- tests and greater than 2-fold change. ^1^Indicates the unique code for the feature on the microarray, ^2^Accession number of the cDNA sequence. ^3^Fold-change for genes higher or lower expressed in fish fed PP relative to fish fed MP diet. ^4^Identity of the probe target as determined by BLASTX and BLASTN searches.

#### Immune and stress response

Regarding the innate immune status, PP fed fish showed lower expression level of inflammatory related genes such as platelet-activating factor receptor, SAMHD1, tumor necrosis alpha-induced protein 2, and the antimicrobial peptide beta defensin 1. Likewise, fish fed the PP diet showed a suppression of genes involved in the adaptive immune response, i.e. TCR β, MHC class I, SMAD 3, CD80 (a T cell activator). Interferon γ induced proteins γ-ip (CXCL10), Gig2-like and both interferon-induced protein 35 and IRF2-binding protein were also lower in fish fed the PP diet. In contrast, Mx and Vig-2 which are responsive to type I interferon were higher expressed in PP compared to MP. Lastly, the stress induced genes HSP 30 and 47 were lower in expression in PP fed fish relative to MP fed fish.

#### Cell proliferation and apoptosis

Genes involved in cell proliferation were lower expressed in the PP fed fish, including ankyrin repeat domain-containing protein 54, nucleostemin and growth hormone-inducible transmembrane protein. Several genes involved in apoptosis were found to be higher such as cell death inducing protein and bh3 interacting domain death agonist although in contrast whereas both caspase 8 and 14 showed lower transcript levels in the PP fed fish.

#### Protein metabolism

Genes involved in protein synthesis such as several 60 S and 40 S ribosomal proteins were lower in expression in skeletal muscle of PP fed fish. A number of enzyme genes that modify essential amino acids (eg serine/threonine-protein kinase PINK1, serine/threonine-protein kinase 35, receptor-interacting serine/threonine-protein kinase 4 amongst others) were also lower in expression. Likewise, a major lysosomal peptidase, cathepsin D was lower in expression in PP fed fish, however, two components of the UbP were higher expressed in PP (ubiquitin-like protein precursor and proteasome subunit beta type-9).

Genes encoding proteins involved in muscle structure and physiology (acta1 protein, actin-binding homolog 1a and tropomyosin-1 alpha chain) were also more highly expressed in skeletal muscle of fish fed the PP diet.

#### Energy metabolism

Genes related to energy metabolism were transcriptionally modified with peroxisomal membrane protein 11 C and cytochrome P450 being higher in expression whereas cytochrome c oxidases (COX2, 8 and 18) were found to be lower in PP fed fish compared to MP fed fish. In addition genes involved in glycolysis (phosphoglycerate kinase 1 and L-lactate dehydrogenase B chain) and gluconeogenesis (malate dehydrogenase 1) were found to be expressed lower in PP fed fish.

### Co-regulated genes

**Figure 2:**
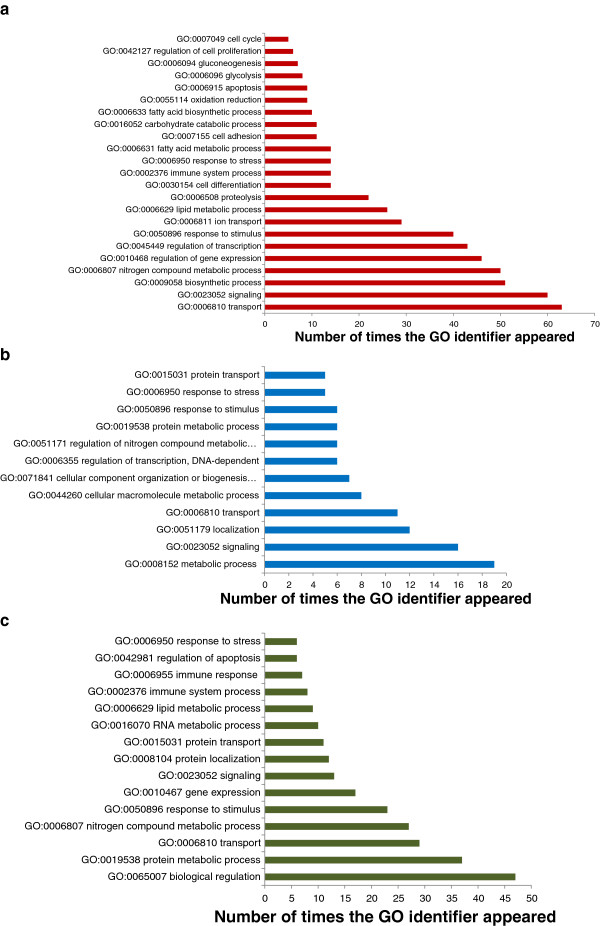
**Gene ontology (biological processes) found to be significantly enriched following the feeding experiment in mid intestine** (**a**)**, liver** (**b**) **and skeletal muscle** (**c**)**.** Only GO categories for which >3 genes were represented are included.

Only a small number of genes were co regulated between tissues following the feeding trial. No genes were significantly higher or lower regulated in all the tissues in this experiment. The greatest co regulation was observed between mid intestine and skeletal muscle, with 9 genes significantly higher and 10 genes significantly lower in PP compared to MP. Genes that were more highly expressed in mid intestine and liver of fish fed the PP diet compared to the MP diet play a role in energy metabolism, such as cytochrome P450, a membrane-associated protein located in the inner membrane of mitochondria and peroxisomal membrane protein 11 C that induces peroxisomal proliferation proteins involved in the pentose phosphate pathway. Genes that showed lower expression in mid intestine and liver have a role in protein synthesis (40 S ribosomal protein S10) and in the immune response, such as SAMHD1 a nuclease involved in innate immune responses by acting as a negative regulator of the cell-intrinsic antiviral response. Five genes were higher expressed in both intestine and liver of fish fed the PP diet, including immune related genes, such as CD209f and MyD88 and only 1 gene (complement c1q) showed lower expression in both tissues. Skeletal muscle and liver did not have any higher expressed gene in common and only 3 genes (membrane-bound O-acyltransferase domain-containing protein 7, SMAD 3 and the non annotated gene (**NP_998306**) were significantly lower between these two tissues (Figure 2; full list of genes expressed at different levels is given in supplementary Table 1).

### Confirmation of expression by real time PCR

**Figure 3:**
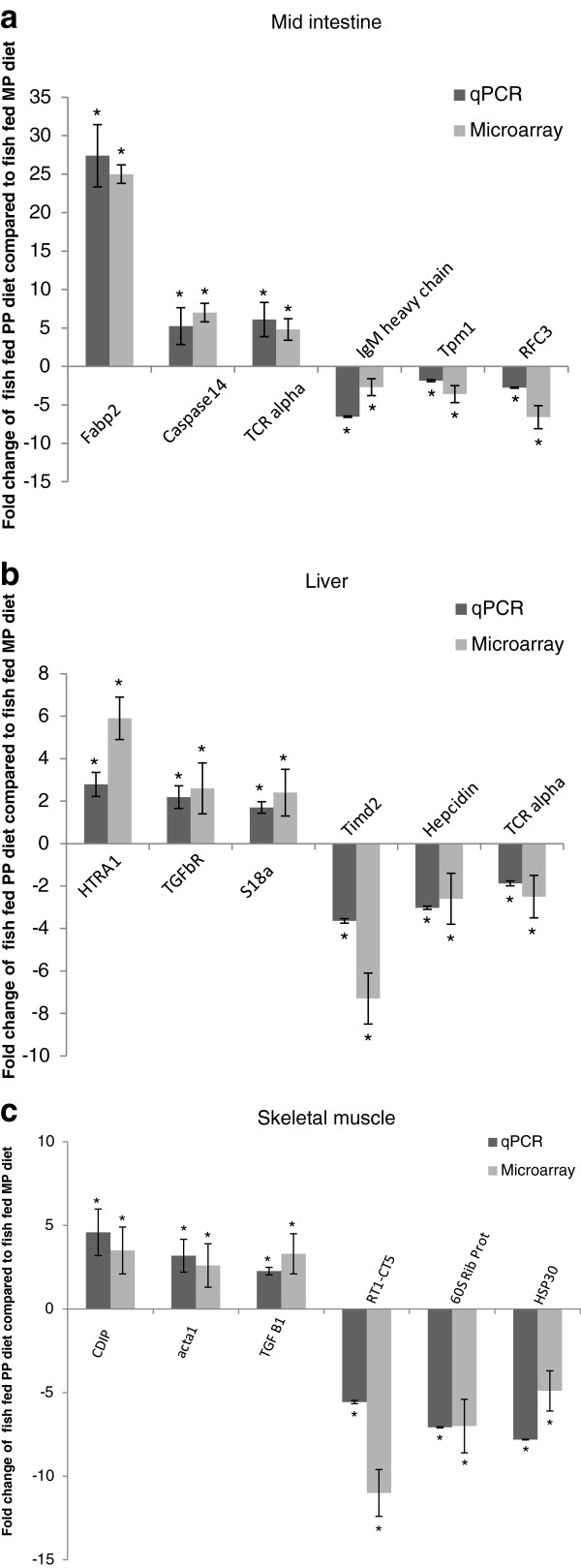
**Quantitative real-time PCR confirmation of genes expressed at different levels in mid intestine (a), liver (b) and skeletal muscle (c) of fish fed the PP diet compared to fish fed the MP diet for 6 genes identified by microarray analysis.** Bars represent mean ± standard error of five fish, asterisks indicate significant (p < 0.05) differences. The genes chosen for the intestine were: Fatty acid-binding protein (Fabp2), Caspase-14 precursor, TCR alpha, IgM heavy chain, Tropomyosin-1 alpha chain (Tpm1) and Replication factor C subunit 3 (RFC3). The genes selected for the liver were: Serine protease HTRA1, TGF beta receptor, 28 S Ribosomal protein S18a (MRPS18A), T-cell immunoglobulin and mucin domain (Timd2), hepcidin I and TRC alpha. The gene chosen for the skeletal muscle were: cell death inducing protein (CDIP), acta1 protein, Transforming growth factor beta-1 (TGF beta 1), MHC class Ib antigen (RT1-CE5), 60Sribosomal protein L6 (60 S rib prot) and Heat shock protein 30.

Real time PCR analysis was performed on a number of genes for each tissue to confirm microarray data (Table 7). The real time PCR expression data was normalized with HPRT1 as the expression of this housekeeping gene was not found modulated by microarray analysis. For all genes the expression pattern showed the same trend between microarray and real time PCR analysis (Figure 3).

**Table 7:** Primers used for real time PCR for expression analysis, Acc is accession number from NCBI

**Gene name**	**Primer name**	**Primer sequence(5-prime to 3-prime)**	**Acc**	**Product size**	**Ann T**	**Tissue**
Fabp2	FatF	GCTCTGTACTAGCTCTCCTCCC	CB509140	156 bp	55 °C	Mid intestine
	FatR	GGCGTACAGTTTGACTATGCAC				
Caspase-14	Cas14F	CGATTATACACCCGGACTATGG	S35582566	155p	55 °C	Mid intestine
	Cas14R	CCTATCAAGTGTGAATCCATGC				
TCR alpha	TCRaF	GGAAGACTCTGCTCTGTACCAC	U50991	147 bp	55 °C	Mid intestine
	TCRaR	GCTGTGGTATTTCTTGACTTC				
IgM heavy chain	IgMF	GCTTATAGCCATAGTACTACTG	S18892409	169 bp	55 °C	Mid intestine
	IgMR	GCATAGCTGCCCCATATCGC				
Tpm1	Trop1F	CGAAGATGAGAGAGATAAAGTGC	TC76471	134 bp	55 °C	Mid intestine
	Trop1R	CCTCCTCAACCAGCTGGATACG				
RFC3	ReplF	GCTGACTCACTGCATTCCTCCTG	S34421775	163 bp	55 °C	Mid intestine
	ReplR	GAAGGCCTCTAGGTGGTAAATGG				
HTRA1	HTRA1F	GGTCATCTTCATACAGAGAGG	S35582821	152 bp	55 °C	Liver
	HTRA1R	GCTTAGAGAATACCATCTTGC				
TGF beta receptor	TGBF	CCACAAGAAGCCAGCTGTCAG	gi|209735249	135 bp	55 °C	Liver
	TGFBR	CTAGCCAGGTATCTCTATCATGG				
MRPS18A	28 S F	CCATTGATTCAGTGAAGCCCATC	DW574268	158 bp	55 °C	Liver
	28 S R	CCTGCTGTGAGTTGACATGC				
Timd2	TcellF	CCATGGACAACCACACACACTG	CA368982	141 bp	55 °C	Liver
	TcellR	CCAGTAGAATGGACACCAGGATC				
Hepcidin I	HepF	GCTTCTGCTGCAAATTCTGAGG	gi|209736931	157 bp	55 °C	Liver
	HepR	GTACAAGATTGAGGTTGTGCAG				
TCR alpha	TCRaF	GGAAGACTCTGCTCTGTACCAC	U50991	147 bp	55 °C	Liver
	TCRaR	GCTGTGGTATTTCTTGACTTC				
CDIP	CellF	CCATGTCTGAGACCTACTCTATG	TC110067	243 bp	55 °C	Skeletal muscle
	CellR	GATAGTCACTTGATGTCCAGTG				
acta1 protein	ActaF	CCTGTAAACTGTGAATGCGTC	TC77227	156 bp	55 °C	Skeletal muscle
	ActaR	CCAAAGTTTTATATCAGCTGC				
TGF beta 1	TGF1bF	GCTCGGAGTGTGAGACAGAACTG	S15319964	187 bp	55 °C	Skeletal muscle
	TGF1bR	CACTTGACGCAACAGAAACACTCC				
RT1-CE5	MHC1bF	GGAAAGATCTCCTGAAGACTTGAG	CK894557	101 bp	55 °C	Skeletal muscle
	MHC1bR	CGTTTATGAGAAGTTCAGC				
60 S rib prot	60SF	GCTTCTTACCATGGTTCTTCAG	DR695852	140 bp	55 °C	Skeletal muscle
	60SR	GGTCAAGATCCCATCCACCATC				
Heat shock protein 30	HeatF	CCATCCAACCAGTCTCCTACAAGC	EG804126	303 bp	55 °C	Skeletal muscle
	HeatR	CCTCCTCAGCAGATAATGGATTC				
Hprt	HprtF	CCGCCTCAAGAGCTACTGTAAT	EG866745	255 bp	55 °C	All tissues
	HprtR	GTCTGGAACCTCAAACCCTATG				

## Discussion

With the continual expansion of aquaculture of piscivorous fish there is a global requirement to reduce the dependence on wild caught fish for the generation of fishmeal and fish oil. There are a number of studies that have explored the transcriptional response to plant and vegetable oil replacements in fish diets, often when 100% replacement experimental diets are used results in reduced growth [24,25]. When both proteins and oils were replaced [24] in rainbow trout microarray analysis of liver indicated processes such as protein metabolism and cell cycle being altered, however the reasons for these changes could either have been combined effect of lowered essential fatty acids or changes in amino acid profile. There is also growing body of work demonstrating the genotype diet interaction in rainbow trout [25-27] and Atlantic salmon [28], with the latter concluding transcription of key metabolic regulators that respond to plant based feeds depend on the fish’s genotype. Commercial salmon feed formulations partially replace fish meal with plant derived proteins, with varying effects on fish physiology and performance including fish growth and food conversion [24,29]. This paper examined the physiological and performance effects with transcriptional responses in mid intestine, liver and skeletal muscle in salmon to a post smolt feed containing high levels of plant derived proteins with low fishmeal content compared to a low plant protein-high fishmeal diet. The tissues examined play different physiological roles in the fish all of which may be affected by dietary changes as reflected in the different response profiles seen in these tissues. In common with a number of other transcriptomic studies on nutritional effects (where extreme dietary manipulations are avoided) the impact of the different diets appears quite subtle suggesting limited changes to physiological and metabolic pathways [30]. Although, in general, the scale of gene response is quite low, distinct changes in all tissues examined do indicate that there are responses occurring as a result of the dietary components.

When interpreting the findings it is important to take a comparative holistic approach whereby apparent down regulation of genes in fish fed one diet may in fact be the result of up regulation in fish fed the other dietary treatment and vice versa. In this paper the expression is analysed relative to the marine profile (MP) diet.

### Histological changes

The occurrence of histological changes was very similar for fish fed either diet, with low frequencies observed for the majority of parameters measured. The main histological observation was a mild fatty change in liver, which is considered normal under intensive rearing conditions. This anomaly appeared to have no clinical effect, and was not prevalent for either dietary group. Although there was a higher incidence of peri‐biliar duct infiltration in liver of fish fed the MP diet no other histological changes were observed to indicate an inflammatory response. This reduced infiltration in the liver by the biliary ducts may be related to the altered immune gene expression in the liver. In mammals viral infections can result in biliary atresia [31] and specifically can be related to immune related injury to bile ducts following infiltration of CD4^+^ T and production of interferon γ [32]. At this stage we are unable to tell if the reduction in the structures is a direct result of the nutritional components or due to the altered hepatic immune gene expression in between the two diets. The distal intestine is often the target organ for anti‐nutriental factors, in particular those present in SBM and pea protein concentrate [22], inducing histological changes including shortening of simple and complex mucosal folds with widening of central stroma, inflammatory cell infiltration in the submucosa and lamina propria with a mixed leukocyte population [33,34]. In this study both dietary groups presented fish with distal intestine anomalies but with low frequencies, and no significant difference between diets. These results together with those reported by Sanden *et al.* (2005) [35] indicate soybean products, including the alcohol soluble fraction, may be used within formulation constraints without inflicting gross histopathological anomalies in the intestine of Atlantic salmon.

### Transcriptome changes in the mid intestine

The fish intestine has multiple functions which will be the first to respond to changes in nutritional intake; particularly digestion and absorption of nutrients and immune responsiveness to ingested pathogens, antigens and new antigens generated by the gut flora via gut associated lymphoid tissue [36]. The intestine contains three distinct regions: the proximal intestine containing pyloric caeca, the mid and distal intestine. Nutrient absorption occurs in all regions via enterocytes, by passive and facilitated diffusion and active transport [37], with the highest rates of uptake in the proximal section [37,38]. The intestine of piscivorous fish can be particularly sensitive to plant derived ANFs and non-starch polysaccharides (NSP) in feed, resulting in altered permeability in trout feed 44% 44% SBM [7,39] and enteritis in the distal intestine of salmon given a high dietary inclusion of SBM [33]. The inflammation/enteritis may be similar to a hypersensitivity reaction [33,40]. Often these effects are temporary and quickly disappear when the intestinal tract is no longer exposed to the ANFs [11,33,41].

Processing of plant products for fish feed is under continual improvement and some current plant derived protein concentrates have very low contaminating factors or botanical impurities. In addition knowledge of ANF containing plant feed materials has increased to the point where commercial feed formulations permit plant derived proteins at acceptable inclusion levels where no negative health effects or impacts on growth and performance occur. This was confirmed in the current study by the histology assessment where no gross morphological changes associated with plant ANFs were observed. In addition there was no difference in growth or feed utilisation efficiency during the feeding period, where a doubling of weight was achieved. However there were more subtle changes to biological processes that were not apparent during the classical physiological evaluation but were detected by global transcriptomic analysis. In this trial the mid intestine showed the greatest transcriptome response of the tissues studied, reflecting the sensitivity of the intestinal cells to dietary factors. Processes modified in the intestine were related to immune parameters, cell proliferation, apoptosis, protein metabolism, energy metabolism, transport and lipid metabolism.

Fish fed the PP diet showed higher expression of genes involved in inflammation suggesting a possible dysfunction in immune regulation. Our findings support previous studies on gut intraepithelial and systemic T cells in fish which showed rainbow trout IELs are rich in T cells [10,42]. Additionally the expression of TSC22D3, a regulator of T cell receptor mediated cell death, was found higher in PP, this protein may be induced by glucocorticoids [43,44] activated by components in the PP diet. Together these results support previous reports that trace levels of substances with allergenic properties may cause expression of genes indicative of a hypersensitivity reaction in the intestine [45].

Interestingly genes involved in the inflammatory response were both higher and lower expressed in PP compared to MP. In particular genes involved in NF-*κ*B pathway were induced such as the signalling adaptor molecule MyD88 and the inhibitory proteins of the IkB family, NF-kB1 p105 which sequesters NF*κ*B in an inactive form in the cytoplasm [46]; inhibition of this pathway results in the production of proinflammatory cytokines [47]. MyD88 is also part of the signalling pathway that induces type I IFNs through the interaction of the MyD88–TRAF6–IRF7 complex [48]. IRF1 [49] had higher expression in fish fed the PP diet, this transcription factor may have activated interferon responsive genes in PP fed fish including Mx [50], virus induced gene (vig) -2 [51] and a virus induced TRIM protein [52]. PP fed fish also had higher expression of (SOCS)-7, which functions to reduce inflammation [53], potentially counteracting the expression of genes related to the inflammatory response in the intestine. Other genes involved in the innate immune response were expressed at lower level in fish fed PP. IL-8 is a chemokine that attracts neutrophils to a site of inflammation [54], whereas IL-17D coordinates the clearance of extracellular bacteria and contributes to the pathology of many autoimmune and allergic conditions in Atlantic salmon [55]. The lower expression of these genes in fish fed the PP diet may indicate there was not a proinflammatory response to the PP diet compared to MP diet.

Several antioxidant genes were expressed higher in PP fed fish mid intestine indicating protection against oxidative damage. The free radicals could be either endogenously produced by immune cells or present in the diet. Alternatively a potential lower concentration of antioxidants in the PP feed, due to the lower fishmeal content, may require the antioxidant system within the fish to be increased accordingly. The overall low expression of heat shock protein mRNAs, suggests a limited stress response in the intestine. Additionally, two genes directly involved in apoptosis process (caspase-3 and14) were expressed at higher level in PP potentially indicating increased apoptotic activity of mid intestinal cells in these fish compared to MP fed fish.

The intestine has an extremely high rate of cellular turnover and hence generally high levels of protein synthesis and protein degradation. Protein metabolism genes relating to both synthesis and degradation were found generally to be higher in PP fed fish suggesting an increase in intestinal protein turnover. Genes related to both transcription and translations were at a higher level such as translation initiation factors, elongation factors and the ribosomal protein S6 kinase. Interestingly, a number of mRNAs encoding ribosomal proteins were at a lower expression in PP. This may relate to the stability of the mRNAs or multiple use of the ribosomal subunits during translation. In parallel to general increase in synthesis genes related to protein degradation were also at a higher level in PP such as cathepsins and components of the ubiqutin proteasome pathway [56,57]. The higher protein turnover is likely to be energy demanding and this is related to an increase in genes encoding proteins involved in oxidative energy metabolism. Together these changes in expression suggest modulation in control of protein turnover in the intestine with components of both synthesis and degradation being altered which may reflect an increased activity of the intestine in fish fed the PP compared to the MP diet.

Cellular membrane transport related genes were higher expressed in PP, which could suggest that salmonids are able to adaptively modulate the densities of transporters to match changes in diet composition. Lipid metabolism and transport were also affected even though the PP diet contained the same fish oil content as the MP diet. mRNAs encoding two apolipoproteins were higher in expression in PP fed fish reflecting a greater mobilization and transport of cholesterol and fatty acids in the intestine, possibly an adaptive response to the lower dietary cholesterol content in PP compared to MP diet. Fatty acid metabolism genes were higher expressed in the PP diet including both FAD5 and 6, a PUFA elongase and other genes related to cholesterol metabolism. FADs are critical enzymes in the biosynthesis of long-chain highly unsaturated fatty acids (HUFA) from shorter chain PUFA [58,59]. These lipid metabolism differences between PP and MP are of interest as the intestine is often over looked regarding these processes and cholesterol, even if the essential fatty acids are present at required levels, other factors including cholesterol may change, revealing the complex nature of the early digestion and modifications of nutrients in the mid intestine.

### Transcriptome changes in the liver

The liver receives nutrients and compounds from the intestine and needs to respond to any substances that may have detrimental effects on the fish.

Transcriptome and proteomic studies on salmonids show nutritionally related modifications in both liver mRNAs and proteins due to feeding status [60,61] and diet composition [9,62-64].

The PP fed fish showed a significant difference to fish fed MP for genes related to immune function with a lower expression of innate factors such as lectins and hepcidin. Acquired immune system components were also found at lower level including T cell receptors, MHC I and II and components of the TGF-β pathway. TGF-β has an important role in the maintenance of T-cell [65] and B-cell homeostasis [66] by regulating cell proliferation process and apoptosis in these cells. This result, with the higher expression of genes involved in cell death such as CIDE-3 and angiopoietin-related protein 4, indicates that apoptosis may be a mechanism induced by the PP diet salmon liver. This is not surprising as apoptosis plays a central role in the differentiation and maintenance of the liver [67]. A balancing effect on the apoptotic TGF-β pathway is seen in the induction of several genes encoding proteins related to cell proliferation (such as HTRA1 serine protease and annexin A3). In particular, HTRA1 serine protease inhibits signalling mediated by TGF-β family proteins [68], playing an important role in contrast to cell death, whereas annexin A3 has a role in the signalling cascade during liver regeneration [69]. Other researchers have found the immune system to be altered following vegetable oil replacement in salmon [28] and in sea bass [70].

Genes involved in oxidative stress response (MPV17 protein, amine oxidase and HSP 70 kDa protein) were higher in liver of fish fed PP compared to MP. This is interesting to note as increases in antioxidant genes were also noted in a salmon diets that had marine oil replaced with vegetable oils [28] in the liver. In rainbow trout HSP expression in liver was increased following SBM rich replacement diets [9,62,63,71], the induction of these genes may indicate a diet-induced stress response in fish fed the PP diet. Moreover, during general high protein turnover to deal with misfolded proteins [72] as may be the case of fish fed PP diet.

It is interesting to note that few lipid related metabolic genes were found significantly different in the liver. Vigilin, a protein implicated in both biosynthesis and metabolism of lipids and steroids, facilitates removal of excess cholesterol from cells [73] and secondly apolipoprotein A IV which facilitates transport of cholesterol to the liver were both expressed at higher level in fish fed PP compared to MP. The high expression of apolipoproteins in fish fed diets containing high levels of plant proteins has previously been observed [9,74] and is most likely indicates reduced cellular cholesterol in fish fed the PP diet due to decreased dietary cholesterol, associated with low fishmeal content, and/or in response to trace levels of phytoestrogens [75] and phorbol esters [76] co purified with the plant proteins.

### Transcriptome changes in the skeletal muscle

Genes related to processes such as protein metabolism, energy metabolism, cell proliferation, apoptosis and immune function were all significantly different in PP fed fish compared to MP. Transcripts related to protein metabolism such as ribosomal protein mRNAs, transcription and translation initiation factors were generally lower in PP fed fish compared to MP fed fish indicating lower protein synthesis. In parallel, protein degradation related genes were also less, for example the ubiquitin proteasome pathway and lysosomal peptidase proteins, together these would suggest a lower protein turnover activity in the muscle tissue in PP group. Both protein synthesis and degradation are highly energy demanding processes [77] and the indication of lower protein turnover, may suggest reduced energy wastage [61,78,79]. This idea is strengthened by lower expression of COX2 and COX8 and other genes encoding proteins involved in glycolysis and gluconeogenesis in fish fed PP. Genes involved in cell proliferation were also expressed at lower levels in PP fed fish indicating further energy saving. Together these changes in biological processes may indicate efficient metabolic activity following feeding of the plant protein enriched diet.

Biological tissues with high metabolic rate require mechanisms to deal with free oxygen radicals, on the other hand those tissues where the metabolic rate is reduced, for example when protein turnover is decreased a reduction in oxidative stress response may be observed as was observed in this study. Additionally a number of HSPs 30, 47 and a heat shock transcription factor 1a were all at lower levels in PP reflecting the reduced protein turnover and requirement of stabilising many newly translated proteins. Genes involved in cell proliferation and related to cell death including two caspases (caspase-8 and 14) were also lower expressed in fish fed PP compared to the MP diet. The induction of bh3 interacting domain death agonist, a pro-apoptotic member of the Bcl-2 protein family [80] and the suppression of caspases is in accordance with apoptosis of skeletal muscle in mammals where the Bcl2/bax system was found crucial for muscle apoptosis, whilst the caspase activity appeared inhibited [81].

Differential expression of a number of immune related genes, particularly a decrease in interferon responsive genes including γ-ip [82], Gig2-like [83] and interferon-induced protein 35 [84] were also expressed at lower level in PP. Other pro-inflammatory agents including platelet-activating factor receptor was also at a lower level PP, which regulates several pro-inflammatory functions such as chemokine and eicosanoid receptors [85]. An antimicrobial peptide beta defensin 1which is a central component of the non-specific defences [86] was also found lower in PP. Relating to the adaptive immune factors, TCRβ, MHCI and CD80 were all at lower levels in PP. Although we have observed differences in genes related to immune function in skeletal muscle of fish fed PP, the low level of inflammation and the subsequent immune response observed in the intestine did not cause a large immune shift in the muscle tissue. Instead, the lower expression of such genes, may allow fish fed PP to spend less energy resources on immune function for use in growth [61].

## Conclusions

In conclusion, the present study confirm that a moderate level of plant protein derived proteins in a salmon diet allowed fish to grow with equal efficiency as those on a high marine protein diet, and with no difference in biometric quality parameters. The PP diet formulated with higher levels of soy protein concentrate, corn gluten, sunflower meal and wheat gluten resulted in significant effects on transcription in the mid intestine, liver, and skeletal muscle. The PP diet induced tissue specific changes in gene expression, with the mid intestine showing activation of the adaptive immune response indicating potential for hypersensitivity and an increase in protein turnover, although no difference in histopathological changes were observed in the proximate, mid or distal intestine. In liver cell proliferation and apoptosis indicate cellular reorganization and the general suppression of processes such as immune response. In contrast skeletal muscle tissue showed reduced protein metabolism and decrease in immune gene expression suggesting less energy expenditure in this tissue. The presence of only few genes in common between tissues may be due to the relatively mild changes that occurred and the complex nature of studying gene expression between tissue types on conservative dietary changes. These results improve the understanding of mechanisms and pathways activated by fishmeal replacement; in particular substitution by plant derived proteins and suggests that such diets can function well in Atlantic salmon aquaculture, hence to some extent reducing the burden on wild caught fish for fish meal. Additionally these results can assist in selection of molecular biomarkers useful for the development of new alternative feeds in salmonid aquaculture.

## Methods

### Fish husbandry and sampling

One hundred and five juvenile mixed sex Atlantic salmon of approximately 100 g were maintained in 6 replicate 1 m^3^ tanks at SGS Chile Ltd., Puerto Mont, Chile, at 10.1°C and 27.6**%** salinity. Fish were fed a plant protein diet (PP) or a high marine protein diet (MP), both of which were formulated to the same digestible protein and energy content within formulation constraints of a commercial feed specification (CPK 100, 3 mm, 24/44 lipid: protein, BioMar S.A., Chile) (Table 1). The feeding trial was conducted in triplicate tanks per dietary treatment and lasted for 77 days. At the end of the feeding period all fish in each tank were bulk weighed and 35 fish from each tank were killed by percussive stunning for individual biometric measurements of round weight, fork length, gutted weight, and weight of liver, heart or intestine for calculation of specific growth rate (SGR), feed conversion ratio (FCR), specific feeding rate (SFR), condition factor, and somatic indices of hepatic (HSI), cardiac (CSI) or intestinal (ISI) organs. Liver, heart and intestine was excised from distinct anatomical regions of the same individuals and fixed in 10% buffered formalin for histological assessment. All fish were sampled 30 minutes following the final meal to ensure there were no differences in postprandial gene expression. An additional 12 fish from each tank were sampled for mid intestine, skeletal muscle and liver and immediately stabilised in RNAlater (Ambion) at 4°C overnight then stored at −20°C until RNA extraction for gene expression analysis.

### Growth/feed parameters were calculated as follows

FCR = feed consumed (g)/biomass increase (g) SGR (% body wt d^-1^) = [(ln*W*_2_ – ln*W*_1_)/*days*] × 100;*W*_1_ = start weight (g), *W*_2_ = final weight (g), *days* = days in the growth periodSFR (%) = SGR × FCRHSI, CSI, ISI (%) = [organ weight (g)/round weight (g)] × 100 Condition factor = [round weight (g) × 100]/[fork length (cm)]^3^Biometric parameters were analyzed for significant differences by Anova using InfoStat v 2009 software (University of Córdoba, Argentina), with p <0.05 considered significant. Data is presented as means ± standard deviation for each dietary treatment.

### Histology assessment

Fixed tissues were submitted to dehydration process following paraffin embedding (Aquagestión, Puerto Montt, Chile). Sections (5 μm) were stained with hematoxylin and eosin (H & E) and examined under a light microscope. Micrographs were examined “blind” by the same experienced pathologist. Liver sections were evaluated for lipid degeneration level (fatty change) and the integrity of the whole organ. Heart tissue was evaluated for the presence of inflammatory infiltrates, myodegeneration and other possible abnormalities. Intestinal morphology was evaluated according to the following criteria: a) widening and shortening of intestinal folds, 2) loss of supranuclear vacuolization in enterocytes in the intestinal epithelium, 3) widening of central lamina propria within the intestinal folds, and 4) infiltration of (mixed) leukocytes in the lamina propria and submucosa [33]. The occurrence of histological changes for each dietary treatment were analysed by Kruskal-Wallis non-parametric Anova with InfoStat v 2009 (University of Córdoba, Argentina), with p < 0.05 considered significant.

### RNA isolation

RNA was extracted from 100 mg of tissue by homogenisation in 1 ml TRIZol (Invitrogen) using tungsten carbide beads (3 mm, Qiagen) and shaking (300 times per min) following the manufacturer’s instructions. The RNA pellet was washed in 500 μl 80% ethanol, air dried and resuspended in RNase free H_2_O. The concentration was determined by spectrophotometry (Nanodrop ND1000, LabTech) and the integrity of the RNA was determined by electrophoresis (Agilent Bioanalyser 2100). The RNA was then stored at −80° until required.

### Microarray analysis

Microarray platform:

Microarray experiments were performed using a custom-designed, Agilent-based microarray platform with 4 × 44 K probes per slide (Salar_2; Agilent Design ID:025520). The array contained primarily an Atlantic salmon resource with 34,441 features from Atlantic salmon coding sequences but additionally a further 9,111 features from rainbow trout (*Oncorhynchus mykiss*) sequences - the latter being selected when no homologues appeared to be available within *Salmo salar* datasets. Full details of the microarray platform and design are shown in Tacchi et al. 2011 [23].

### Hybridization and analysis

For microarray analysis, 4 pools of RNA were produced for each tissue from fish fed PP and MP diets. Each RNA pool was an equimolar RNA mix from four different fish chosen randomly from each group. The microarray hybridisation was performed using a reference design, using a reference RNA sample, which comprised an equimolar mix of RNA extracted from all individual fish and tissue samples. Each experimental sample (labelled with Cy3^TM^) was hybridised against this reference sample (labelled with Cy5^TM^) in a 2-colour experiment. mRNA amplification, labelling and hybridization was performed as follows: mRNA was amplified using a MessageAMP^TM^ aRNA Amplification Kit (Ambion). Briefly, 2 μg total RNA was reverse transcribed and the cDNA was used as a template for *in vitro* transcription in the presence of amino allyl modified dUTP, which allowed the generation of amplified antisense RNA (aRNA). For labelling, aRNA (3 μg) was denatured at 70°C for 2 min in a volume of 10 μl to which 3 μl of 0.5 M NaHCO_3_ and 2 μl Cy dye (dye Cy3^TM^ or Cy5^TM^ mono-reactive dye pack, Amersham) was added. Incorporation of dyes was performed for 1 h in the dark, and after excess label was removed using a DyeEx^tm^2.0 spin kit (QIAGEN) the amount incorporated was checked with a Nanodrop ND1000 (LabTech) spectrophotometer. Prior to hybridisation, 825 ng of each labelled template was fragmented in the presence of 11 μl of 10X blocking agent, 2.2 μl of 25X Fragmentation buffer (Agilent), and made up to a final volume of 20 μl with nuclease-free dH_2_O. The solution was then incubated in the dark at 60°C for 30 min, after which 57 μl of 2X GEx Hybridisation buffer (Agilent) was added to each sample and 103 μl of each hybridisation solution was dispensed on the Agilent 4x44K Atlantic salmon “*Salmo salar2*” oligo array (Agilent array design, 025520, Array express platform A-MEXP-1940). The hybridisations were performed in a Microarray Hybridisation Oven (Agilent) overnight (18 h) at 65°C. Following hybridisation, the slides were washed in Gene Expression Wash buffers 1 and 2 (Agilent) following the manufacturer’s instructions. The slides were then scanned using a GenePix personal 4100A Scanner (Axon Instruments) at a resolution of 5 μm and saved as *. TIF files. Images were extracted and initial analysis was performed by Feature extraction v9.5.3 (Agilent) performing background correction of feature intensities (within the software). A Lowess normalisation of background corrected data was next conducted and all intensity values <1.0 were set to 1.0. Statistical analysis of the arrays was performed using Genespring GX analysis platform (version 9.5; Agilent Technologies). Quality control of the data was performed within Genespring and included removal of saturated probe features, non-uniform features, population outliers and those features showing intensities not significantly different from background in the Cy3 or Cy5 channels. After these relatively stringent procedures, 20,095 of the original 43,730 array features were maintained for subsequent analyses. The experimental hybridisations are at European Bioinformatics Institute archived under accession number E-MEXP: E-TABM-1207.

Significant differential expression between fish fed the PP diet and fish fed the control diet was established by *t*-test analysis (*p* < 0.05). Further filtering on fold change was conducted, and only transcripts showing more than two-fold change in expression were further characterised.

### Analysis of gene ontology

Enrichment for gene ontology (GO) biological processes was performed on all cDNA features that had GO identifiers associated using the GOEAST program [87]. Fisher’s exact test was performed within the GOEAST program to determine if GO identifiers occurred more often in a group than would appear by chance. For GO analysis only biological process GO identifiers were considered that occurred more than 3 times.

### Real time PCR

The cDNA was synthesized using 2 μg of total RNA as previously described [88]. Real time PCR was performed on a number of genes to confirm the microarray analysis results using the same RNA samples. For cDNA synthesis, 2 μg of total RNA was denatured (70°C, 3 min) in the presence of 1 μl of oligo-dT_17_ (500 ng μl^-1^), left at room temperature for 5 min to allow annealing, then stored on ice. The resulting cDNA was diluted to a final volume of 50μl in RNA/DNA free water (Sigma). For real time PCR, 3μl of cDNA was used as template with gene specific primers (Table 7). A 2x iQ SYBR Green supermix (Bio-Rad) was used for qPCR, which was performed in a 96-well plate using the DNA Engine OpticonTM system (MJ Research, Inc.) with the following program: 95°C for 5 min, then 35 cycles of 94°C for 30° s, 55°C for 30 s and 72°C for 30 s, with a final extension of 72°C for 5 min. A negative control (no template) reaction was also performed for each primer pair. A sample from the serial dilution was run on a 2% agarose gel and stained with ethidium bromide and viewed under UV light to confirm a band of the correct size was amplified. A melting curve for each PCR was determined by reading fluorescence every degree between 72°C and 95°C to ensure only a single product had been amplified. Atlantic salmon hypoxanthine phosphoribosyl transferase 1 (HPRT1) [89] was used as control for normalization of expression since this gene was found not to be modulated by the diet treatments from the microarray analysis. The relative expression level of the genes was determined using the Pfaffl method [90]. Efficiency of the amplification was determined for each primer pair using serial 10 fold dilutions of pooled cDNA, performed on the same plate as the experimental samples. The efficiency was calculated as E = 10 ^(−1/s)^ where s is the slope generated from the serial dilutions, when Log dilution is plotted against ΔCT (threshold cycle number). Primers were design to have a Tm of 55°C, and where possible, to cross an exon-exon junction sites to avoid amplification of genomic DNA. Exon-intorn junction sites were determined comparing *Salmo salar* cDNA with genomic sequences for orthologous genes from *Danio rerio*, *Gasterosteus aculeatus*, *Oryzias latipes*, *Takifugu rubripes* and *Tetraodon nigroviridis* obtained from Ensembl ( http://ensembl.org/).

The results obtained by real time PCR were analyzed using the Pfaffl method [90]. The qPCR measurements were analyzed by *T*-test, performed using R software, with p < 0.05 considered significant. The expression data is presented as means ± standard error.

## Competing interests

The authors declare that they have no competing interests.

## Author’s contributions

LT performed microarray experiments, analyzed the data, carried out the real time PCR and wrote the manuscript. MAC, CV and HT were involved in fish maintenance, feeding trial and sampling. SAMM, CJS, RB and MAC were involved in the experimental design and drafting of the manuscript. All the authors read and approved the final manuscript.
